# Supplementary figures and images for: The potential of UAV-borne spectral and textural information for predicting aboveground biomass and N fixation in legume-grass mixtures
Source: PLoS One. 2020 Jun 25;15(6):e0234703. doi: 10.1371/journal.pone.0234703 (PMC7316270; doi:10.1371/journal.pone.0234703)

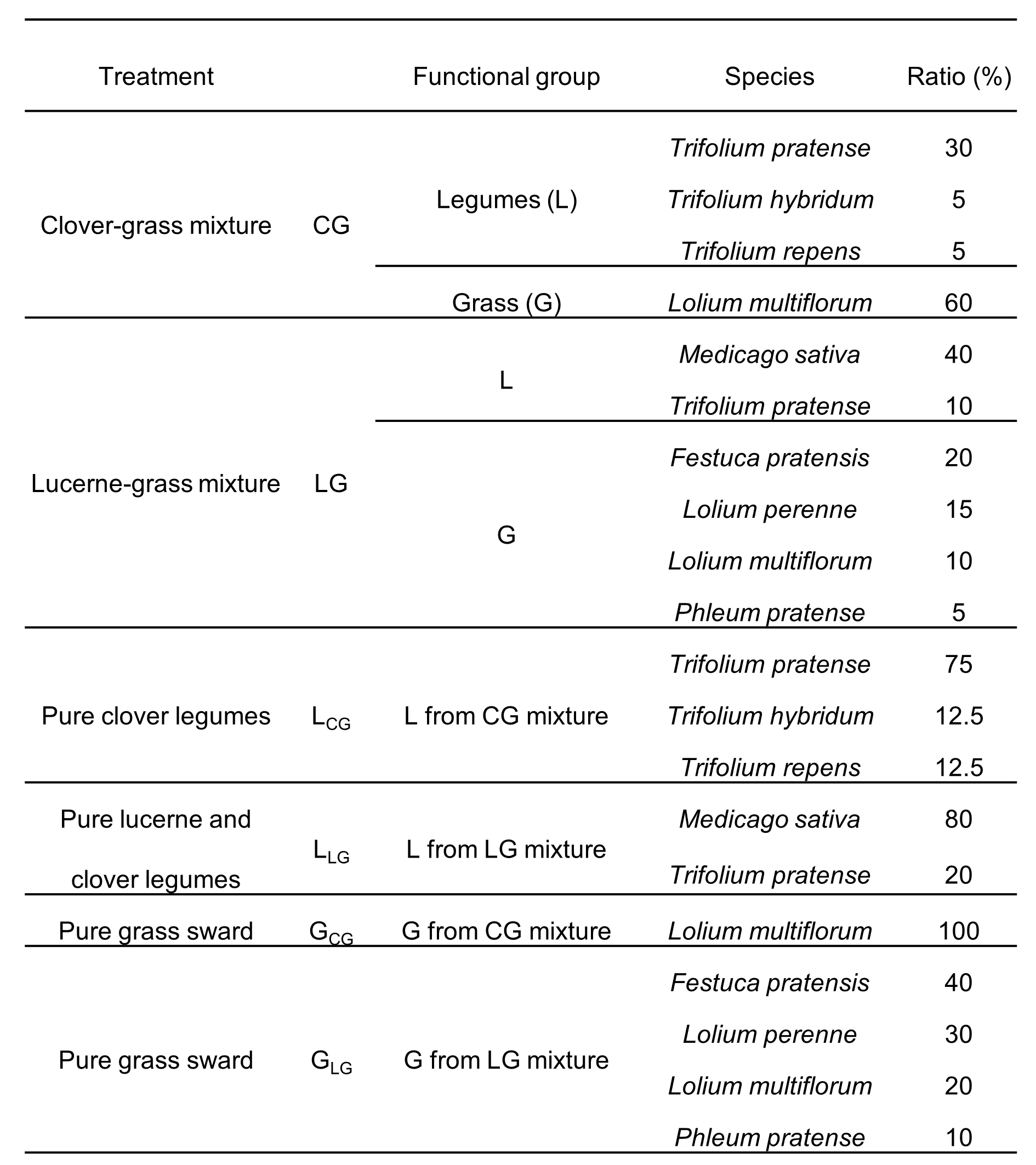

Supplement: S1 Table — CG = Clover-grass; LG = Lucerne-grass. (TIF) [file pone.0234703.s001.tif]

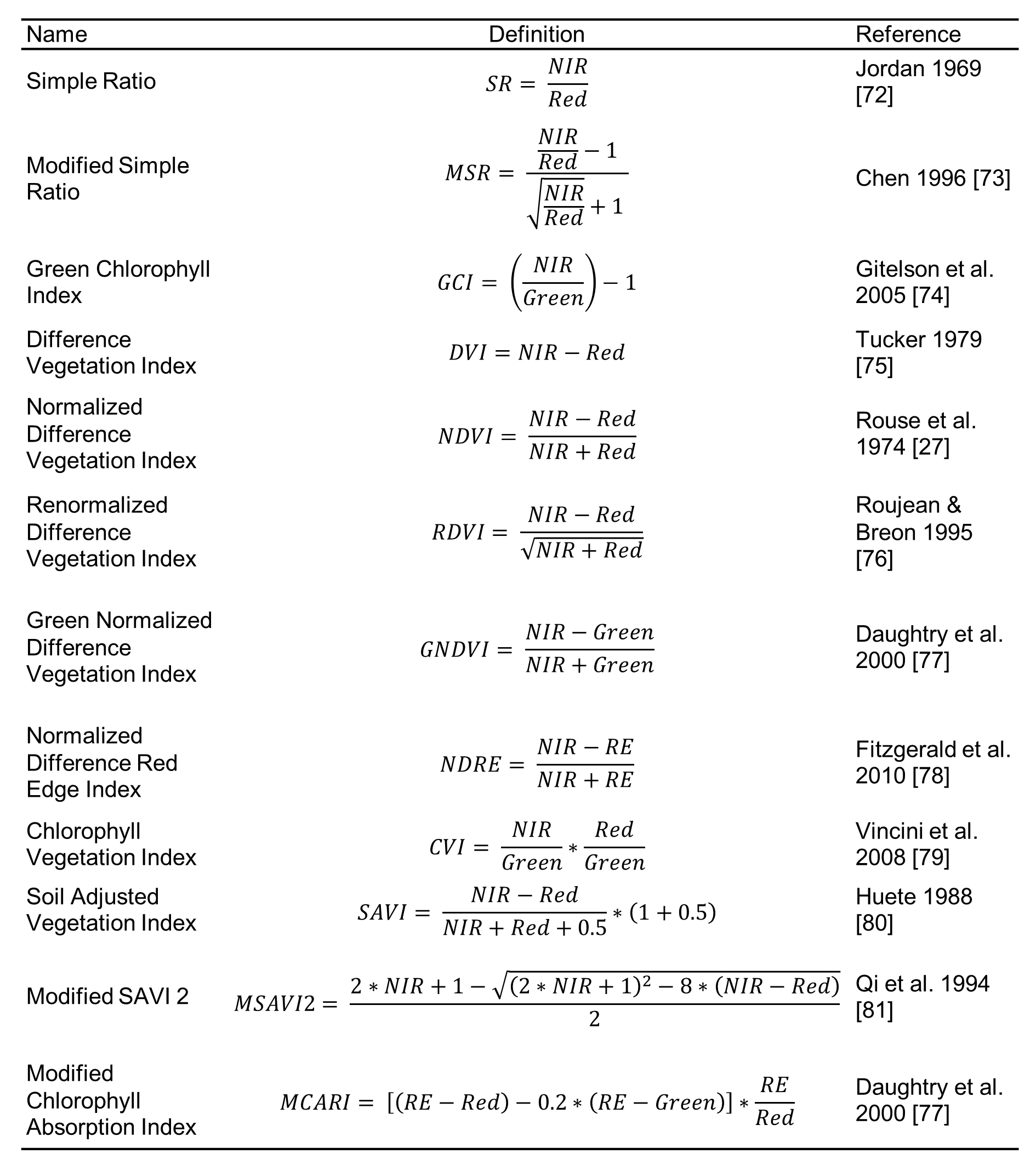

Supplement: S2 Table — Vegetation indices calculated with four bands captured by the multispectral sensor used in this study: green, red, red edge (RE) and near infra-red (NIR). (TIF) [file pone.0234703.s002.tif]

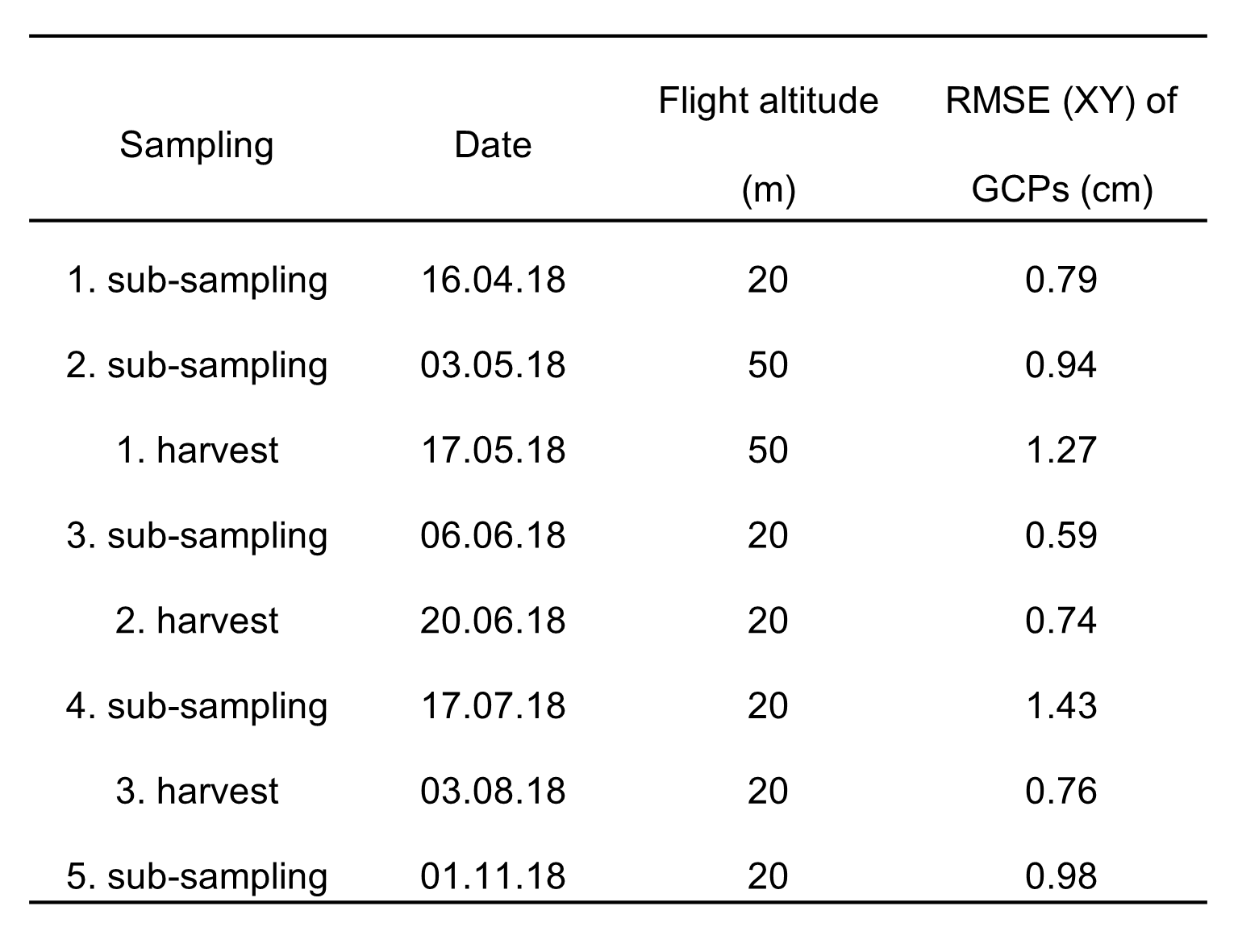

Supplement: S3 Table — (TIF) [file pone.0234703.s003.tif]

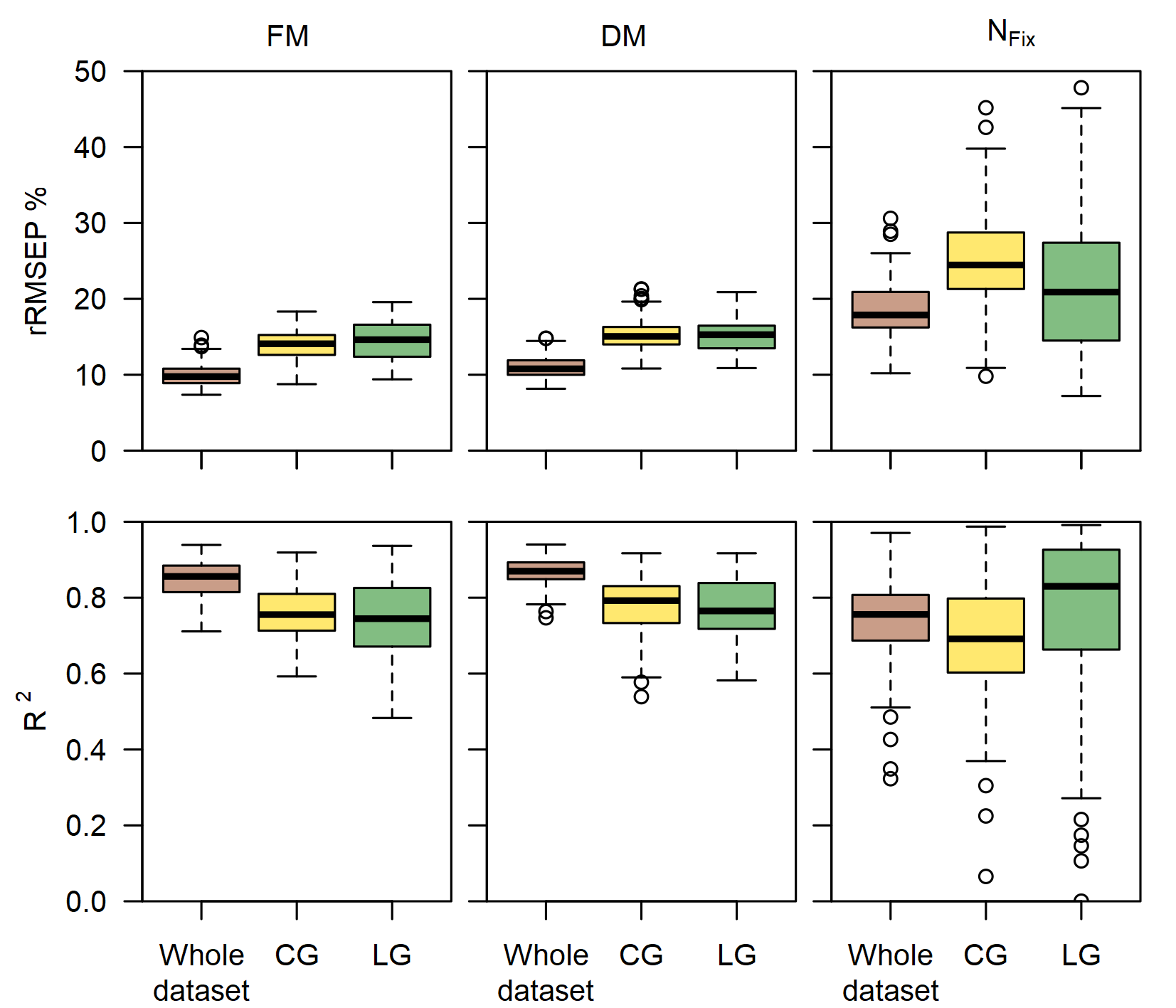

Supplement: S1 Fig — Boxplots for the model accuracy created by 100 cross-validations for the whole dataset as well as crop-specific: clover-grass (CG) and lucerne-grass (LG) mixtures including the pure stands of legumes and grass. Plots show the best prediction algorithm, with 100 randomly selected test and training data sets based on data from 3 main harvests and 6 sub-sampling dates, whereas NFix contains only main harvests. Boxes show the 25 and 75% percentile, the solid line indicates the median, the whiskers represent the 5 and 95% percentile, circles show outliers. (TIFF) [file pone.0234703.s004.tiff]

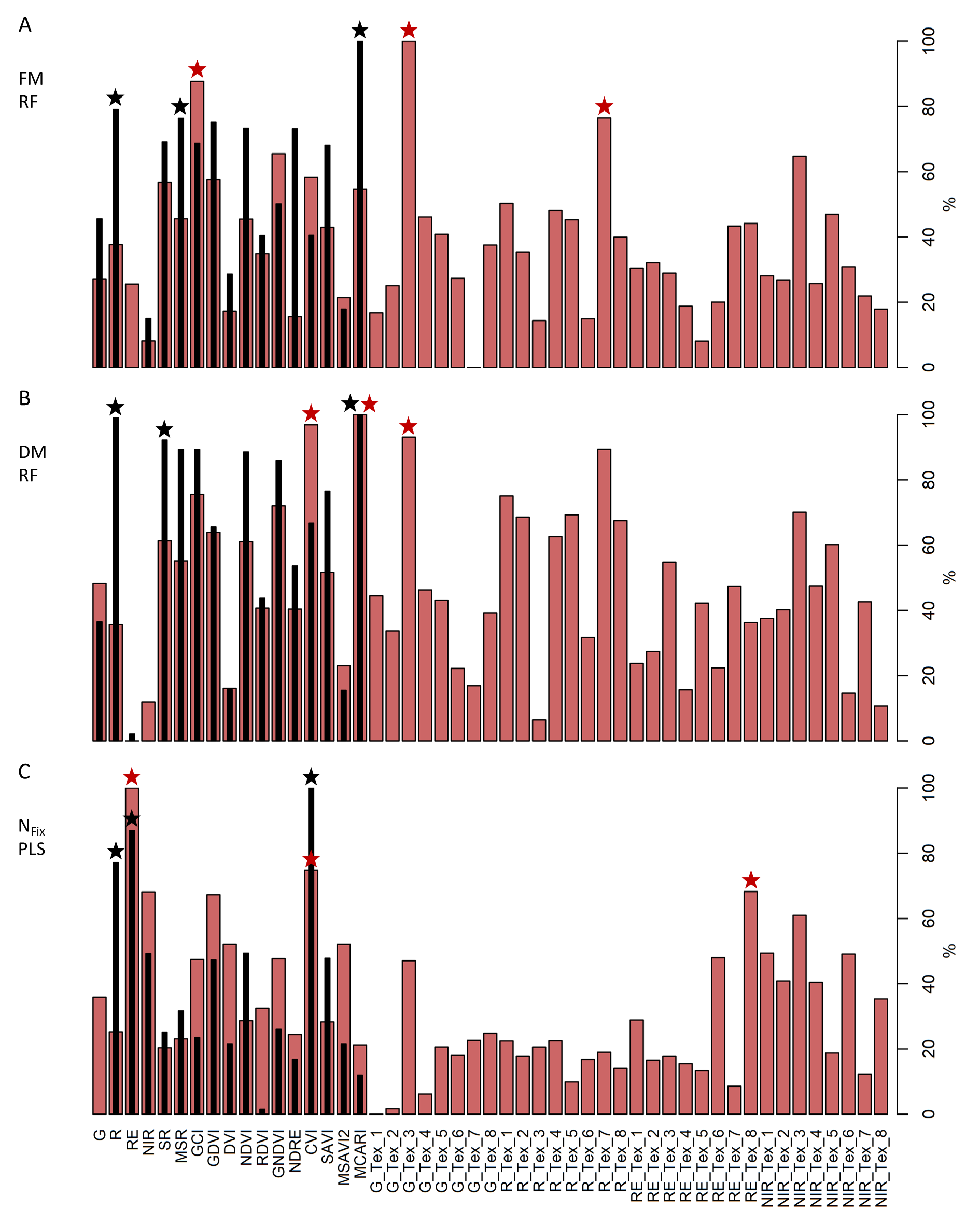

Supplement: S2 Fig — Variable importance of prediction models for fresh (FM) (A) and dry matter (DM) (B) as well as fixed N (NFix) (C) for the whole dataset built with four spectral bands, 13 vegetation indices and with (red) and without (black) 8 texture features of each band. Stars indicate the three highest rankings of variables with (red) and without texture features (black) in the model. Plots show the best prediction algorithm, Partial Least Square (PLS) or Random Forest (RF), with the best of 100 randomly selected test and training data sets based on data from 3 main harvests and 6 sub-sampling dates, whereas NFix contains only main harvests. (TIF) [file pone.0234703.s005.tif]

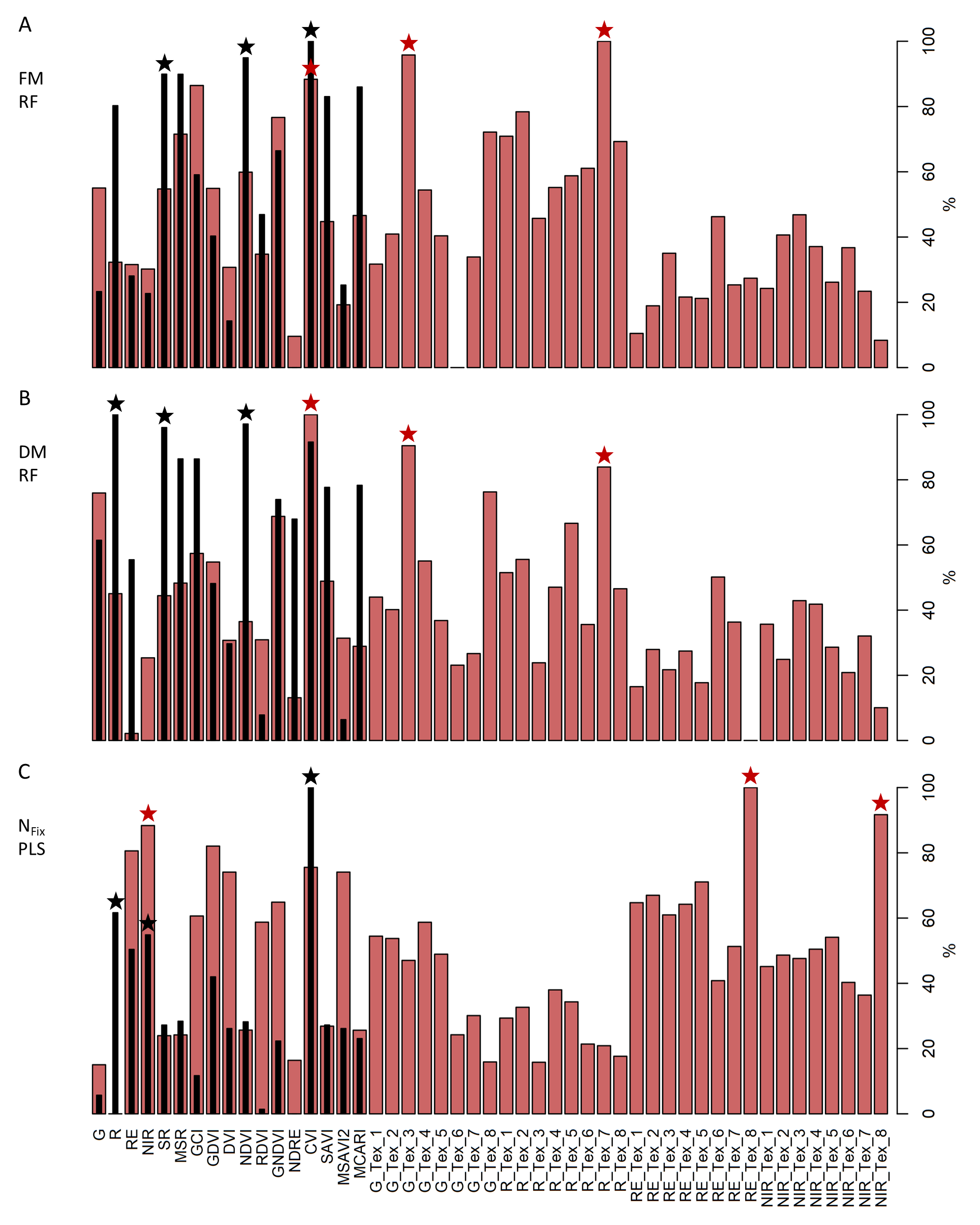

Supplement: S3 Fig — Variable importance of prediction models for fresh (FM) (A) and dry matter (DM) (B) as well as fixed N (NFix) (C) for the whole dataset built with four spectral bands, 13 vegetation indices and with (red) and without (black) 8 texture features of each band. Stars indicate the three highest rankings of variables with (red) and without texture features (black) in the model. Plots show the best prediction algorithm, Partial Least Square (PLS) or Random Forest (RF), with the best of 100 randomly selected test and training data sets based on data from 3 main harvests and 6 sub-sampling dates, whereas NFix contains only main harvests. (TIF) [file pone.0234703.s006.tif]
