# Supplementary material for: The potential of UAV-borne spectral and textural information for predicting aboveground biomass and N fixation in legume-grass mixtures
Source: PLoS One. 2020 Jun 25;15(6):e0234703. doi: 10.1371/journal.pone.0234703 (PMC7316270; doi:10.1371/journal.pone.0234703)
Supplement: Attachment — Submitted filename: Response to reviewers.pdf [file pone.0234703.s008.pdf]

Manuscript ID: PONE-D-19-34750R1 - Response letter

Dear Mrs. Müllerová,

Dear Reviewers,

thank you very much for the second review of our manuscript titled „ The potential of UAV-borne spectral and textural information for predicting aboveground biomass and N fixation in legume-grass mixtures". We have carefully implemented the comments.

Please find our specific answer to each reviewer's comments below in **bold letters**.

Yours sincerely

Esther Grüner

Reviewers' comments:

Reviewer #1: I really appreciate the work the authors have been performed in order to critically reflect and significantly have improved the research presented. From my point of view all comments have been clearly addressed and I recommend this contribution for publication.

Thank you.

Reviewer #2: Dear Authors,
it is true that a flight height of 20-50 m and a limited short flight duration (and small area) can suggest to do not apply any atmospheric correction. However my concern was about the processing of a time series of images. From one image time acquisition to another the atmospheric influence could be different. And this can affect the VI values. Please just shortly discuss this topic and how this could affect your analysis.

We have carefully implemented this comment to L. 572-577 in the section "Discussion".
