# Supplementary material for: The potential of UAV-borne spectral and textural information for predicting aboveground biomass and N fixation in legume-grass mixtures
Source: PLoS One. 2020 Jun 25;15(6):e0234703. doi: 10.1371/journal.pone.0234703 (PMC7316270; doi:10.1371/journal.pone.0234703)
Supplement: Attachment — Submitted filename: Response to reviewers.docx [file pone.0234703.s007.docx]

**Manuscript ID: PONE-D-19-34750 - Response letter**

Dear Mrs. Müllerová,

Dear Reviewers,

thank you very much for the review of our manuscript titled „ The potential of UAV-borne spectral and textural information for predicting aboveground biomass and N fixation in legume-grass mixtures “, which greatly helped to improve it. We have carefully implemented the comments.

Due to modification of the data processing (as proposed by Reviewer 1), major changes were made in section “UAV image acquisition and data pre-processing” (from L: 222), “Prediction models” (from L: 403) and minor changes in section “Discussion” (from L: 578).

Providing metadata (140 data point with 52 variables) and eight orthomosaics to the supplements of this manuscript seems not operable to us. This data will be part of the published data in a public repository.

Please find our specific answer to each reviewer’s comments below in **bold letters**.

Yours sincerely

Esther Grüner

PLOS ONE

Journal Requirements:

1. When submitting your revision, we need you to address these additional requirements.

Please ensure that your manuscript meets PLOS ONE's style requirements, including those for file naming. The PLOS ONE style templates can be found at

http://www.journals.plos.org/plosone/s/file?id=wjVg/PLOSOne_formatting_sample_main_body.pdf and http://www.journals.plos.org/plosone/s/file?id=ba62/PLOSOne_formatting_sample_title_authors_affiliations.pdf

2. Our internal editors have looked over your manuscript and determined that it is within the scope of our Plant Phenomics & Precision Agriculture Call for Papers. This collection of papers is headed by a team of Guest Editors for PLOS ONE. The Collection will encompass a diverse range of research articles spanning disciplines, methods and applications. Additional information can be found on our announcement page: https://plos.io/phenomics.

If you would like your manuscript to be considered for this collection, please let us know in your cover letter and we will ensure that your paper is treated as if you were responding to this call. If you would prefer to remove your manuscript from collection consideration, please specify this in the cover letter.

**Yes, we would like our manuscript to be considered for Plant Phenomics & Precision Agriculture Call for Papers. Thank you.**

3. We note that you have stated that you will provide repository information for your data at acceptance. Should your manuscript be accepted for publication, we will hold it until you provide the relevant accession numbers or DOIs necessary to access your data. If you wish to make changes to your Data Availability statement, please describe these changes in your cover letter and we will update your Data Availability statement to reflect the information you provide.

**Metadata and orthomosaics will be published at gfbio (German Federation for Biological Data: www.gfbio.org), which is in progress.**

4. Please include your tables as part of your main manuscript and remove the individual files. ** Please note that supplementary tables (should remain/ be uploaded) as separate "supporting information" files **.

**Done.**

[Note: HTML markup is below. Please do not edit.]

Reviewers' comments:

Reviewer's Responses to Questions

Comments to the Author

1. Is the manuscript technically sound, and do the data support the conclusions?

The manuscript must describe a technically sound piece of scientific research with data that supports the conclusions. Experiments must have been conducted rigorously, with appropriate controls, replication, and sample sizes. The conclusions must be drawn appropriately based on the data presented.

Reviewer #1: Partly

Reviewer #2: Yes

________________________________________

2. Has the statistical analysis been performed appropriately and rigorously?

Reviewer #1: Yes

Reviewer #2: Yes

________________________________________

3. Have the authors made all data underlying the findings in their manuscript fully available?

The PLOS Data policy requires authors to make all data underlying the findings described in their manuscript fully available without restriction, with rare exception (please refer to the Data Availability Statement in the manuscript PDF file). The data should be provided as part of the manuscript or its supporting information, or deposited to a public repository. For example, in addition to summary statistics, the data points behind means, medians and variance measures should be available. If there are restrictions on publicly sharing data—e.g. participant privacy or use of data from a third party—those must be specified.

Reviewer #1: No

Reviewer #2: Yes

________________________________________

4. Is the manuscript presented in an intelligible fashion and written in standard English?

PLOS ONE does not copyedit accepted manuscripts, so the language in submitted articles must be clear, correct, and unambiguous. Any typographical or grammatical errors should be corrected at revision, so please note any specific errors here.

Reviewer #1: Yes

Reviewer #2: Yes

________________________________________

Reviewer #1: 1. Is the manuscript technically sound, and do the data support the conclusions?

Overall, the manuscript is technical sound and an interesting contribution to the field. The authors have performed a thorough data capture of field data and reference data as well. I would to suggest to review the following sections in order to provide a clarification:

Materials and Methods

A lot of data have been captured (multispectral UAS, Field Reference, GCP) and a lot of different variables have been computed and statistically analyzed. It would be very helpful to provide a summary table about all available data sets, calculated variables (VI, GLCM) and performed regression analysis.

**The authors agree, that a lot of different variables were included in the analysis. Therefore, a workflow (Fig. 2) for a better overview was designed and implemented to the section “Experimental site and ground truth data” (L. 299).**

165: Please make sure that the full experimental design necessary and relevant in order to follow your argumentation is summarized in this manuscript. It seems to me that this is described between 165 -174, please review this issue.

**The authors ensured that all relevant information of the experimental design is included in section “Experimental site and ground truth data”. Minor changes were done (L. 173).**

189 +: Fig. 1: From this figure it is not clear, how B (i.e. the design of the experimental Layout) can be seen in A? I would suggest to clearly indicate the layouts from B in A.

**The authors decided to keep two separate figures for a better overview. The figure (Fig. 1, L.: 188) has been modified for a better understanding how B is included in A by white borders of the plots in A.**

UAV Image acquisition and data preprocessing.

This chapter has significant potential for improvement. It is not clear why the UAS was flown manually and not using a predefined flight mission plan. This brings a certain level of uncertainty regarding data quality of UAS imagery as you always have different flight geometries. Furthermore, it is not clear why there has been a change of flight altitude between the first 2 flights and the 3rd flight resulting in different GSD’s for the different flights. Please argue, why flight 3 was included in the analysis as I assume a change in resolution will also affect for example the calculation of GLCM?

**There are eight flight missions in total and two of them are done with a flight altitude of 50 m, whereas the remaining five are at 20 m. For these two flight missions the image overlap was 100%. Therefore, as a compromise between flight height and time, remaining cuts were flown at 20 m. All flight missions were flown manually as by removing the original camera, automatic flight missions were not possible due to internal technical problems. This information was included from L. 249 to 255. All flights were included in the analysis to keep data size high. This has only minor effects on spectral information, but the authors agree that it can affect texture analysis. Future work should focus on unified flight heights, which is now mentioned in section “Discussion” (L. 697).**

In order make the photogrammetric processing transparent, I suggest to add as supplementary information the metadata from the processing reports, i.e. in particular the overlap, and also the RMSE as the authors have used GCP’s.

**Done (S3 Table, L.: 965). The information about the number of overlapping images is given in L. 263.**

In general, please add a comment what is the desired GSD in order to capture the desired grassland properly. How is this reflected in selecting the flight altitude in combination with sensor resolution.

**GSD had minor effects on multispectral information. Our aim was to keep the flight missions feasible and, therefore, it was a compromise between flight time and resolution. This was implemented in L. 250.**

Please describe also, if, and if yes, how you performed a resampling as each UAS orthomosaic has a different resultion – what was here your final target resolution in order perform a proper coregistration of the individual images from each mission.

**The authors thank the editor for this comment and agree, that a resampling is necessary. The orthomosaics were resampled to a unified resolution of 4.5 cm (L. 280). Consequently, all analysis was repeated and results changed minor.**

It would also strengthen your manuscript, if you can provide the orthomosaics for all three missions.

**As there are eight orthomosaics in total (three harvests and five sub-sampling dates), such attachment would not substantially improve information in this manuscript. We suggest instead, that the orthomosaics will be part of the published data in a public repository.**

Data analysis and machine learning

245: Please explain, what do you mean by “avoiding the first 1.5 m”. In 167 you describe the size of a field plot is 1.5mx12m – if avoid 1.5 m – what is then left from the plot? Please clarify! Maybe a figure showing your analysis design in detail might help?

**To avoid disturbance of the sub-sampling in regrowth of biomass for the main harvests, the sub-sampling was restricted to the first 1.5 m (Fig. 1 B, light red area). Therefore, the remaining area for the analysis was 15.75 m^2^. This information was added in L. 290.**

A zonal statistic has been performed calculating the mean of each variable. I would suggest to provide here a basic statistic description of the variables including MIN, Max, STDV etc. At least I would suggest to add here a comment on variability of the individual variables and the potential of loosing information due to averaging?

**The authors agree, that by averaging variable values information may get lost. Nevertheless, by including texture features, spatial information is still part of the analysis. Due to the size and complexity the authors would like to avoid adding the statistical description (MIN, MAX, STDV etc.) for the 52 variables included in the study.**

Texture features of images

251-252: Please explain, what is the rationale behind selecting 8 textural features out of the 14 as suggested by Haralick? Is there any evidence which of selected 8 is suitable for grassland texture? Please justify/argue/clarify your selection of texture features.

**As the utilization of texture features was never done before in biomass estimation for grassland or legume-grass mixtures, there was no evidence for selecting certain texture features. This study was a first approach to using such information at all. This is emphasized in L. 307. In the future, a more systematic selection of the most important texture features may improve or simplify the biomass estimation models.**

Vegetation index calculation

Please argue, why you have selected the mentioned 13 VI?

**We used VIs which could be created with the available 4 bands and which were mentioned in literature for biomass estimation of vegetation and grassland. This was clarified in L. 322.**

2. Has the statistical analysis been performed appropriately and rigorously?

Yes.

3. Have the authors made all data underlying the findings in their manuscript fully available?

Not for the review.

4. Is the manuscript presented in an intelligible fashion and written in standard English?

Yes.

158: ……located in northern Hessen.

**(L. 163) The authors disagree. In English the German state “Hessen” is called Hesse (though it sounds weird).**

Reviewer #2: The manuscript is very well organised and the English style is good. The topic is interesting for the potential impact on operational precision farming. I have just few concerns:

1) It seems that the multispectral images were calibrated in reflectance using the irradiance sensor installed on the UAV. If this is true, then your images were calibrated in reflectance at platform level and not in ground reflectance. Since you are processing a time series of images this could be an important aspect to discuss. Also VI can be affected by the atmosphere, depending on their formulation. I think that this argument deserves a discussion;

**The authors agree, that the atmosphere between sensor and ground can affect reflection. Nevertheless, flight height of 20-50 m was rather low and, thus, are the atmospheric effects on the reflectance. Additionally, it can be assumed that within the study area and given a very short flight time no fluctuation of the atmospheric conditions appeared. An atmospheric correction is rather used for satellite data and large areas, where atmospheric variation within the study area can be expected. Therefore, the authors would prefer to not discuss this in the manuscript.**

2) In you analyses you are using the 13 VI as independent variables. However I would like to stress that these VI are usually correlated and for sure they are correlated with the original bands values. Please confirm or not that this is not a problem for the validity of your application, from statistical point of view.

**To deal with multispectral data, which usually suffers from multicollinearity, machine learning algorithms are a solution. This method is commonly used in the field of remote sensing also with grasslands. For a better understanding, this is emphasized in L. 341.**

Pag 17 - please check this sentence "The radiometric resolution was set to 0 to 255."

**(L. 309) Changed to 16 bits.**
